# A retroperitoneal cavernous hemangioma arising from the gonadal vein: A case report

**DOI:** 10.1097/MD.0000000000022325

**Published:** 2020-09-18

**Authors:** Chun-Yo Laih, Po-Fan Hsieh, Guang-Heng Chen, Han Chang, Wei-Ching Lin, Chun-Ming Lai, Chao-Hsiang Chang

**Affiliations:** a Department of Urology; b School of Medicine; c Graduate Institute of Biomedical Sciences, School of Medicine, China Medical University; d Department of Pathology; e Department of Radiology, China Medical University Hospital; f Department of Computer Science, Tunghai University, Taichung, Taiwan.

**Keywords:** case report, cavernous hemangioma, gonadal vein, retroperitoneal

## Abstract

**Rationale:**

Cavernous hemangioma (CH) is not commonly found within the abdomen or the retroperitoneum. We report the first case of CH originating from the gonadal vein.

**Diagnosis:**

A retroperitoneal tumor was found incidentally in a 57-year-old female patient. The differential diagnoses from the initial imaging studies included gastrointestinal stromal tumor, carcinoid tumor, neurogenic tumor, metastasis, lymphadenopathy, or another rare tumor.

**Interventions:**

A surgical en-bloc excision was performed via a subcostal incision and intravenous CH arising from a gonadal vein was diagnosis by the urological pathologist.

**Outcomes:**

After the surgery, no complications were noted. A computed tomography scan was performed after 3 months follow-up and no tumor recurrence was found.

**Lessons:**

This case reminds us that CH should be listed as one of the differential diagnoses for a retroperitoneal tumor. A definite diagnosis of CH relies on surgical resection. The prognosis is well if adequate resection is performed.

## 1 Introduction

Cavernous hemangiomas (CHs) are benign endothelial cell neoplasms that are typically absent at birth. However, it may grow rapidly during infancy with spontaneous involution later in life.^[1,2]^ CHs are most commonly seen in the orbital areas but are not commonly observed within the abdomen of the retroperitoneum. CHs that originate in the retroperitoneum are even rarer.^[3–6]^ Retroperitoneal CHs tend to be asymptomatic, especially in the early stages of their development. However, if they grow large enough to compress the adjacent anatomical structures, the patient starts to get symptoms. The radiographic features of CHs are diverse and may be observed using ultrasonography, computed tomography (CT), angiography, and magnetic resonance imaging (MRI).^[7]^ Thus, it is hard to make a definitive diagnosis for retroperitoneal CH through any non-surgical method. Here we share the case of a 57-year-old female patient who had CH arising from the gonadal vein.

## 2 Case presentation

**Figure 1:**
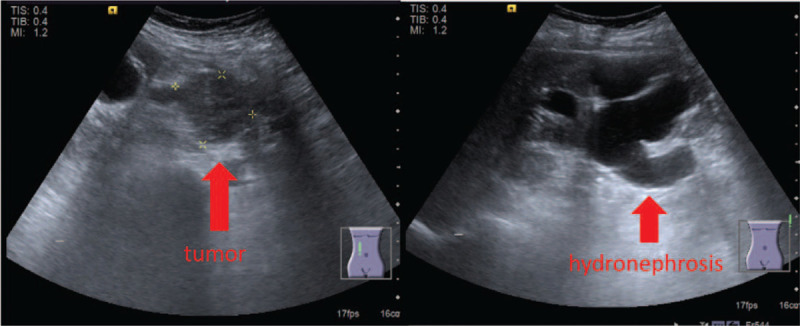
A heterogeneous hypoechoic lesion measuring 4.5 × 3.9 cm was found over the right retroperitoneal cavity by ultrasound, and induced right moderate hydronephrosis.

**Figure 2:**
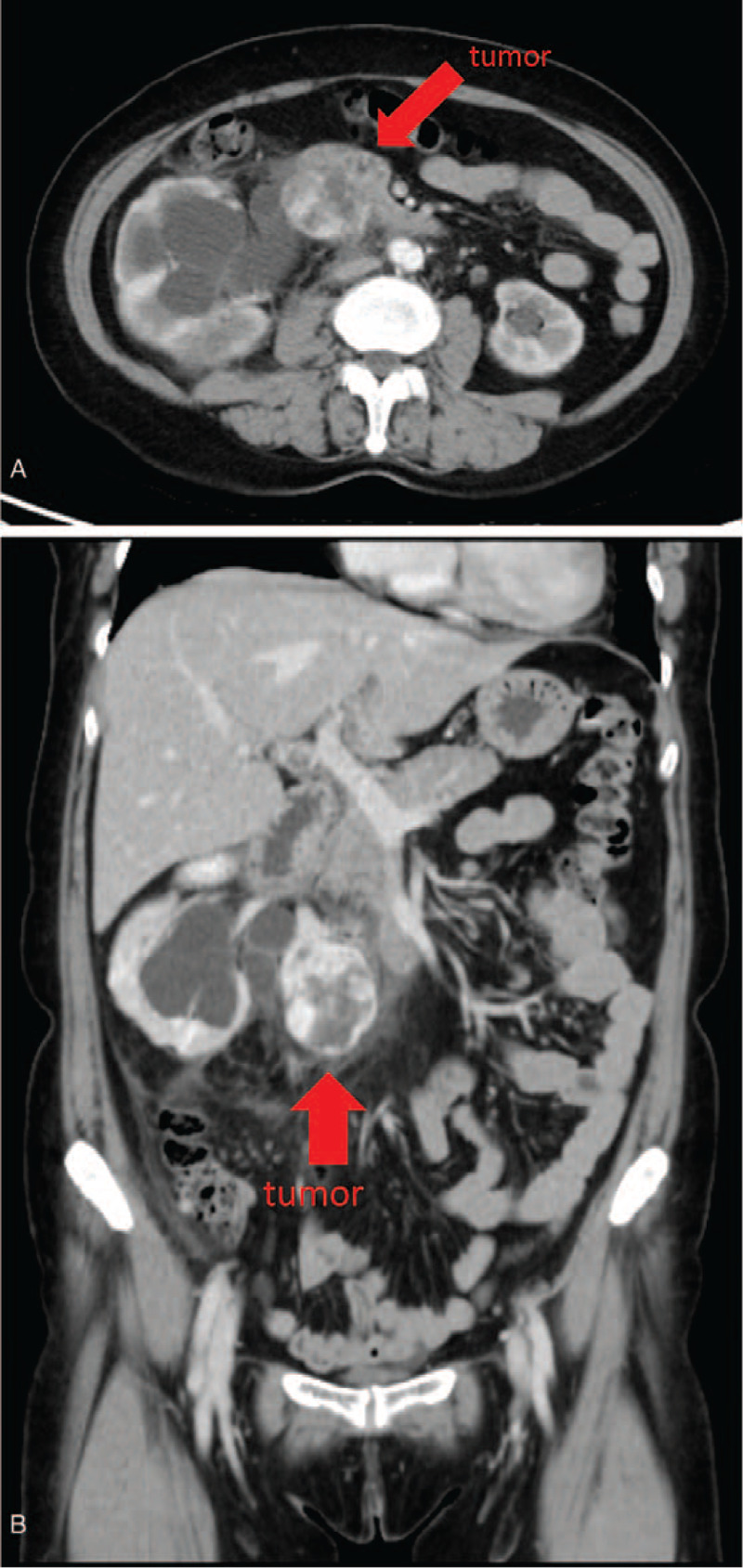
CT scan showed heterogeneous enhancement over the right retroperitoneum. (A) Transverse view, (B) coronal view. CT = computed tomography.

This study presents the case of a 57-year-old female patient who had a history of hypertension, diabetes mellitus, and left breast cancer post a modified radical mastectomy in April 1989. The patient gave informed consent for the publication of her case. She was transferred to our urological clinic because of an incidentally identified heterogenous tumor in the right retroperitoneal cavity, which was 4.5 × 3.9 cm in size, and causing right hydronephrosis (Fig. 1). A subsequent CT scan (Fig. 2A and B) revealed heterogeneous enhancement over the right retroperitoneum. The differential diagnoses included gastrointestinal stromal tumor (GIST), carcinoid tumor, neurogenic tumor, metastasis, lymphadenopathy, or another rare tumor. No remarkable symptoms were noted by the patient prior to surgery. Laboratory studies, including tests for serum creatinine, amylase, lipase, bilirubin, alanine, aspartate aminotransferases, and urine analysis were all within normal ranges. We suggested a further MRI scan, but the patient asked for surgical intervention as soon as possible.

We created a subcostal incision, and the retroperitoneal tumor was found medial to the right upper third ureter, lateral to the duodenum, and above the inferior vena cava. One draining vein into the right renal vein, and another 2 feeding arteries arising from the renal artery were noted. It was not possible to clearly identify the gonadal vessel within the operative field. In addition, a slight adhesion was noted between the tumor, hydroureter, and second portion of the duodenum area.

**Figure 3:**
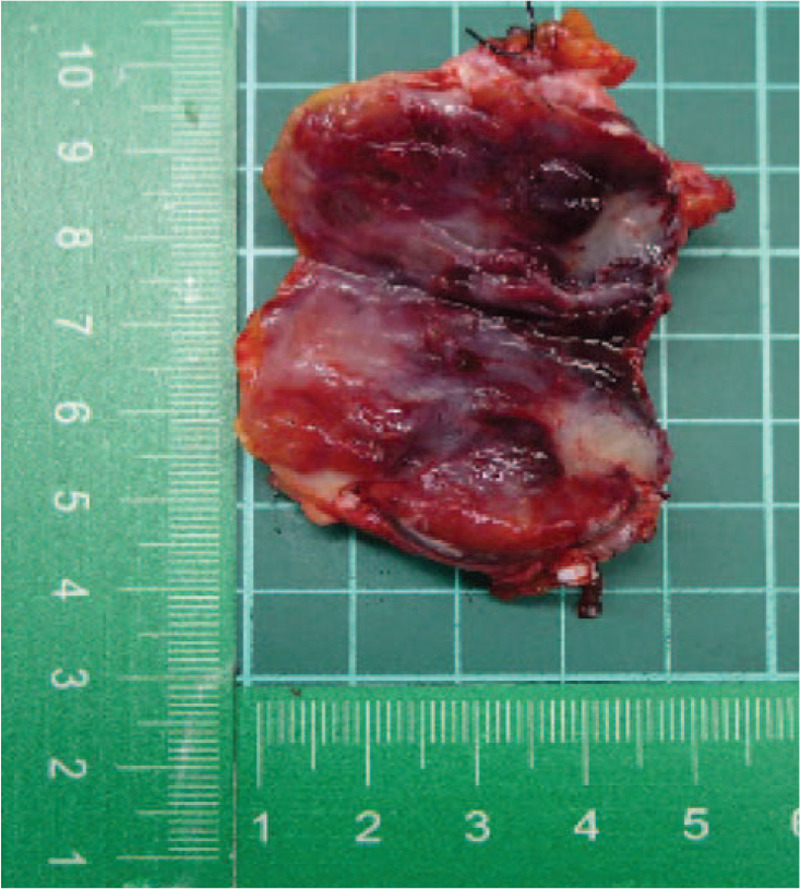
(A) and (B): Gross examination of the resected specimen.

**Figure 4:**
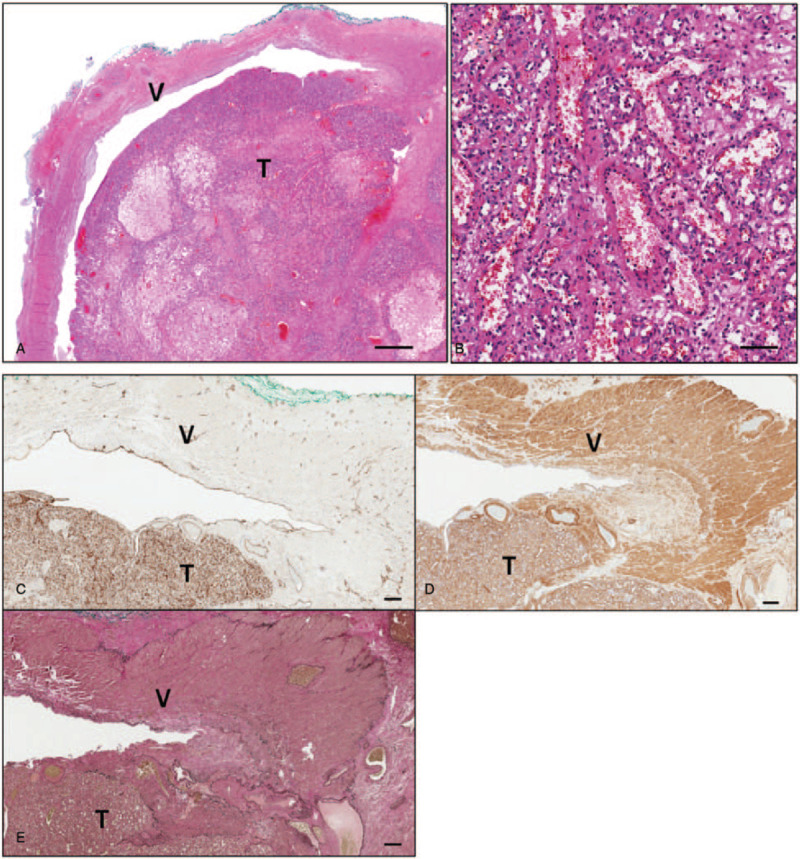
(A) The cavernous hemangioma arising from the venous wall, and protruding into the venous lumen and extending into the adventitia. V, vein; T, tumor; scale bar, 1 mm. (B) High magnification shows the tumor composed of variable-sized benign capillaries and small venules. Scale bar, 100 mm. (C) CD31 immunoreactivity to the vascular endothelia of the veins and capillaries in the tumor. V, vein; T, tumor; scale bar, 200 μm. (D) Actin immunoreactivity to the vascular walls of the veins and capillaries in the tumor. V, vein; T, tumor; scale bar, 200 μm. (E) Elastic-van-gieson staining shows the black elastic fibers in the venous wall, confirming the cavernous hemangioma arising from the venous wall. V, vein; T, tumor; scale bar, 200 μm.

Gross examination of the resection specimen (Fig. 3) revealed a tan and elastic tumor consisting of a tissue fragment with focal hemorrhaging and an elastic consistency. Microscopically, the sections revealed a vascular tumor, composed of lobulation of proliferating capillaries within a delicate or loose fibrous stroma. This vascular tumor involved the large venous wall, as demonstrated by actin immunostaining and Elastic-van-gieson staining, and adjacent soft tissue. This picture is compatible with a diagnosis of intravenous CH arising from a large vein (gonadal vein). CD31 immunostaining exhibited diffuse positivity on the endothelium of these vascular channels (Fig. 4).

No complications were noted after the surgery and the patient was discharged after 5 days. A CT scan was performed after 3 months follow-up and no tumor recurrence was found.

## 3 Discussion

Vascular tumors are non-epithelial and can be divided into hemangioma and vascular malformations. CH is a benign vascular malformation often seen in the orbital, skin, and mucosal areas. However, it is rarely found within the retroperitoneal cavity.^[7,8]^ CH is usually observed in infants and children but seldom in adults. The probability of getting CH is similar for both sexes.^[7,9–11]^ There are no specific symptoms for patients who have abdominal or retroperitoneal CH. Flank pain, symptomatic or asymptomatic hematuria, anemia, thrombocytopenia, renal vein thrombosis (rarely), and even life-threatening bleeding have been reported as symptoms of renal CH.^[12]^ In the current case, the patient did not have flank pain despite hydronephrosis. Maybe slow progression of retroperitoneal CH is the reason for this.

The most common sites for retroperitoneal CH include the peripancreatic, perirenal, or periureteric locations, and the iliopsoas muscle area. The kidney is the most common site of genitourinary CH, followed by the urinary bladder.^[13,14]^ Within the kidney, the most common site is the renal papilla.

Retroperitoneal CH is very difficult to diagnose before surgery. In this case, the differential diagnosis included GIST, carcinoid tumor, neurogenic tumor, metastasis, lymphadenopathy, or another rare tumor. GIST was the first diagnosis of the uro-radiologist because of the heterogeneous enhancement. According to the literature, CH may have features of homogeneous enhancement relative to the renal cortex.^[7,10]^ It may also present as a homogeneous, hypodense inner component tumor without enhancement during the artery or portal phase. The reason for atypical findings on the CT scan might be neovascularity, hemorrhage, thrombosis, and arteriovenous shunting.^[7]^ An MRI did help with the diagnosis of retroperitoneal CH. On T1 imaging they usually show low signal intensity. Whereas on T2-weighted imaging, hemorrhage, and hyalinization of tissue can often be interpreted with high intensity within the tumor.^[15–17]^ Although we did offer the option of a MRI examination before the operation, the patient declined and instead surgery was performed after a physical exam.

Retroperitoneal hemangioma is very rare and only confirmed in <5% of all retroperitoneal tumors in the adult. Less than 30 cases had been reported since 1950.^[17,18]^ Thus, there are no golden standard therapeutic option to follow. The generally recommended treatment for retroperitoneal hemangioma was surgical resection.^[7,17]^ Even though retroperitoneal hemangioma is a benign tumor, patients still have the risk of tumor rupture and bleeding without monitoring nor treatment. As for the prognosis, no obvious complication nor recurrence are found in these cases in follow-up, although local recurrence had been reported sporadically if primary resection was inadequate.^[17]^

In conclusion, this case reminds us that CH should be listed as one of the differential diagnoses for retroperitoneal tumors. The definite diagnosis of CH relies on surgical resection. The prognosis is well if adequate resection is performed.

## Author contributions

**Conceptualization:** Chun-Yo Laih, Guang-Heng Chen, Han Chang, Wei-Ching Lin.

**Writing – original draft:** Chun-Yo Laih, Chun-Ming Lai.

**Writing – review & editing:** Chun-Yo Laih, Po-Fan Hsieh, and Chao-Hsiang Chang.

## Abbreviations

Abbreviations: CH = cavernous hemangioma, CT = computed tomography, GIST = gastrointestinal stromal tumor, HU = Hounsfield unit, MRI = magnetic resonance imaging.

## References

[1] Haik BG Karcioglu ZA Gordon RA. Capillary hemangioma (infantile periocular hemangioma). Surv Ophthalmol 1994;38:399 426. 8009426 10.1016/0039-6257(94)90172-4

[2] Rosca TI Pop MI Curca M. Vascular tumors in the orbit--capillary and cavernous hemangiomas. Ann Diagn Pathol 2006;10:13 9. 16414539 10.1016/j.anndiagpath.2005.07.008

[3] Korumilli R Reddy G. A rare case of retroperitoneal cavernous hemangioma. Int Surg J 2014;1:37 8.

[4] Matsuda D Iwamura M Baba S. Cavernous hemangioma of the adrenal gland. Int J Urol 2009;16:424. 19416406 10.1111/j.1442-2042.2009.02260.x

[5] Weidenfeld J Zakai BB Faermann R. Hemangioma of pancreas: a rare tumor of adulthood. Isr Med Assoc J 2011;13:512 4. 21910381

[6] Geenen RW Den Bakker MA Bangma CH. Sonography, CT, and MRI of giant cavernous hemangioma of the kidney: correlation with pathologic findings. AJR Am J Roentgenol 2004;182:411 4. 14736672 10.2214/ajr.182.2.1820411

[7] He H Du Z Hao S. Adult primary retroperitoneal cavernous hemangioma: a case report. World J Surg Oncol 2012;10:261 4. 23216883 10.1186/1477-7819-10-261 PMC3539936

[8] England RJ Woodley H Cullinane C. Pediatric pancreatic hemangioma: a case report and literature review. JOP 2006;7:496 501. 16998249

[9] Takaoka E Sekido N Naoi M. Cavernous hemangioma mimicking a cystic renal cell carcinoma. Int J Clin Oncol 2008;13:166 8. 18463963 10.1007/s10147-007-0700-z

[10] Mundinger GS Gust S Micchelli ST. Adult pancreatic hemangioma: case report and literature review. Gastroenterol Res Pract 2009;2009:839730 5. 19421421 10.1155/2009/839730 PMC2676326

[11] Kieger AJ Nikolaidis P Casalino DD. Adrenal gland hemangioma. J Urol 2011;186:2415 6. 22019159 10.1016/j.juro.2011.09.067

[12] Zhao X Zhang J Zhong Z. Large renal cavernous hemangioma with renal vein thrombosis: case report and review of literature. Urology 2009;73:443.e1 3. 10.1016/j.urology.2008.02.049 18407336

[13] Ojili V Tirumani SH Gunabushanam G. Abdominal hemangiomas: a pictorial review of unusual, atypical, and rare types. Can Assoc Radiol J 2013;64:18 27. 22397826 10.1016/j.carj.2011.08.004

[14] Jahn H Nissen HM. Haemangioma of the urinary tract: review of the literature. Br J Urol 1991;68:113 7. 1884135 10.1111/j.1464-410x.1991.tb15276.x

[15] Mossanen M Dighe M Gore J. Large retroperitoneal hemangioma encompassing the renal vein. Can Urol Assoc J 2015;9:E894 6. 26834900 10.5489/cuaj.3356 PMC4707912

[16] Igarashi J Hanazaki K. Retroperitoneal venous hemangioma. Am J Gastroenterol 1998;93:2292 3. 9820421 10.1111/j.1572-0241.1998.00642.x

[17] Hanaoka M Hashimoto M Sasaki K. Retroperitoneal cavernous hemangioma resected by a pylorus preserving pancreaticoduodenectomy. World J Gastroenterol 2013;19:4624 9. 23901241 10.3748/wjg.v19.i28.4624 PMC3725390

[18] Braasch JW Mon AB. Primary retroperitoneal tumors. Surg Clin North Am 1967;47:663 78. 6022980 10.1016/s0039-6109(16)38243-3

